# A Novel Biomimetic Approach to Repair Enamel Cracks/Carious Damages and to Reseal Dentinal Tubules by Amorphous Polyphosphate

**DOI:** 10.3390/polym9040120

**Published:** 2017-03-25

**Authors:** Werner E. G. Müller, Maximilian Ackermann, Meik Neufurth, Emad Tolba, Shunfeng Wang, Qingling Feng, Heinz C. Schröder, Xiaohong Wang

**Affiliations:** 1 European Research Council-Advanced Investigator Grant Research Group at the Institute for Physiological Chemistry, University Medical Center of the Johannes Gutenberg University, Duesbergweg 6, D-55128 Mainz, Germany; mneufurt@uni-mainz.de (M.N.); emad_nrc@yahoo.com (E.T.); wsfwmkx@googlemail.com (S.W.); hschroed@uni-mainz.de (H.C.S.); 2 Institute of Functional and Clinical Anatomy, University Medical Center of the Johannes Gutenberg University, Johann Joachim Becher Weg 13, D-55099 Mainz, Germany; maximilian.ackermann@uni-mainz.de; 3 Key Laboratory of Advanced Materials of Ministry of Education of China, School of Materials Science and Engineering, Tsinghua University, 100084 Beijing, China; biomater@mail.tsinghua.edu.cn

**Keywords:** amorphous polyphosphate microparticles, retinyl acetate, enamel cracks/fissures, *Streptococcus mutans*, human mesenchymal stem cells, collagen type I, alkaline phosphatase

## Abstract

Based on natural principles, we developed a novel toothpaste, containing morphogenetically active amorphous calcium polyphosphate (polyP) microparticles which are enriched with retinyl acetate (“a-polyP/RA-MP”). The spherical microparticles (average size, 550 ± 120 nm), prepared by co-precipitating soluble Na-polyP with calcium chloride and supplemented with retinyl acetate, were incorporated into a base toothpaste at a final concentration of 1% or 10%. The “a-polyP/RA-MP” ingredient significantly enhanced the stimulatory effect of the toothpaste on the growth of human mesenchymal stem cells (MSC). This increase was paralleled by an upregulation of the MSC marker genes for osteoblast differentiation, *collagen type I* and *alkaline phosphatase*. In addition, polyP, applied as Zn-polyP microparticles (“Zn-a-polyP-MP”), showed a distinct inhibitory effect on growth of *Streptococcus mutans*, in contrast to a toothpaste containing the broad-spectrum antibiotic triclosan that only marginally inhibits this cariogenic bacterium. Moreover, we demonstrate that the “a-polyP/RA-MP”-containing toothpaste efficiently repairs cracks/fissures in the enamel and dental regions and reseals dentinal tubules, already after a five-day treatment (brushing) of teeth as examined by SEM (scanning electron microscopy) and semi-quantitative EDX (energy-dispersive X-ray spectroscopy). The occlusion of the dentin cracks by the microparticles turned out to be stable and resistant against short-time high power sonication. Our results demonstrate that the novel toothpaste prepared here, containing amorphous polyP microparticles enriched with retinyl acetate, is particularly suitable for prevention/repair of (cariogenic) damages of tooth enamel/dentin and for treatment of dental hypersensitivity. While the polyP microparticles function as a sealant for dentinal damages and inducer of remineralization processes, the retinyl acetate acts as a regenerative stimulus for collagen gene expression in cells of the surrounding tissue, the periodontium.

## 1. Introduction

Teeth, as complex organs, are composed of two separate specialized hard tissues, dentin and enamel; they form an integrated attachment unit with bone via the specialized, periodontal ligament. Teeth are ectodermal organs that develop through sequential and reciprocal interactions between oral epithelial cells (ectoderm) and the cranial neural crest derived mesenchymal cells. From the epithelial cells, ameloblasts form the enamel, while the mesenchymal cells form all the other differentiated cells, such as the dentin forming odontoblasts. Postnatally, teeth continue to develop: the outer layer giving rise to enamel continuously becomes harder, while dentin and root formation start to occur as part of tooth eruption [1]. Like bone, teeth enamel and dentin are composed of biogenic apatite, but lack cells. Enamel and dentin are primarily composed of hydroxyapatite (HA) crystals. Enamel almost exclusively consists of an inorganic matrix (96% (*w*/*w*)) with only very little organic constituents (proteins and lipids) and of water (4%, (*w*/*w*)); the latter components occupy the gaps along the apatite crystals. In enamel the HA crystals have a hexagonal morphology and are bundled to form ≈4 µm diameter rods. In contrast, mature dentin contains only about 70% (*w*/*w*) mineral; the rest is 20% (*w*/*w*) organic matrix, and 10% (*w*/*w*) water [2]. The morphology of the HA crystals in dentin is different and comprises flattened plates, ≈60–70 nm in length, 20–30 nm width, and 3–4 nm thickness. The overall calcium and phosphorus (as phosphate) content of the teeth ranges between 34% to 39% (*w*/*w*) and 16% to 18% (*w*/*w*), respectively. In addition, other cations and anions are incorporated into cationic (Ca^2+^) and anionic centers (OH^−^, PO_4_^3−^) of the HA matrix; Na^+^, K^+^ and Mg^2+^ can substitute the Ca^2+^ position, F^−^ and Cl^−^ can replace OH^−^ and CO_3_^2−^ can substitute both the OH^−^ and the PO_4_^3−^ positions [3,4].

In contrast to bone and dentin, mature enamel is acellular and cannot regenerate itself. Two, perhaps promising, strategies for enamel re-growth have been proposed. First, a chemical synthesis of enamel-like HA nanorods (organized bundles of apatite crystals) was published. However, in order to provide this material with a suitably sufficient affinity to the physiological enamel, surfactant systems had to be tried [5]. In a second biomimetic approach, the production of enamel-like material has been attempted by in situ remineralization of enamel [6] in the presence of amelogenin, enamelin, and/or enzymes [7]. It had been the aim to initiate in a controlled process a defined crystal initiation to generate crystal shape, and packing organization like in enamel. It had been proposed that those peptides allow the de novo enamel crystal formation [8] and simultaneously a decrease demineralization [9].

The mineralization pathway to crystalline HA starts from enzymatically formed amorphous Ca-carbonate [10] that is transferred non-enzymatically to amorphous Ca-phosphate (ACP) [11] which is subsequently processed to the crystalline Ca-phosphate state, HA (reviewed in: [12]). Recent data indicate that P_i_, released from the natural polymer polyphosphate (polyP) through enzymatic, exohydrolytical cleavage via alkaline phosphatase (ALP) acts as a supply of P_i_ during bone formation [13,14]. This polymer is present in considerable amounts in the circulating blood serum, as well as within cells, especially within blood platelets (reviewed in: [15]). In a new biomimetic approach we prepared amorphous polyphosphate (a-polyP) by *co*-precipitating of Na-polyphosphate with CaCl_2_ in the presence of poly(ethylene glycol) [16]; under those conditions microparticles with a size range of 150 to 250 nm are formed. Na-polyP in general and a-polyP in particular have been found to be morphogenetically active due to the property of the polymer to elicit the expression of those genes that are involved in cell growth activation and cell differentiation, e.g., ALP and bone morphogenetic protein-2 (BMP-2), as recently reviewed [12,13,14]. Furthermore, in a recent study, we recognized that a-polyP microparticles form a tight interaction especially with the dentin of human teeth [17]. This result in concert with the in vivo result showing that polyP, entrapped in PLGA (poly(d,l-lactide-*co*-glycolide))-based microspheres, accelerate bone regeneration in calvarial critical-sized defects faster compared to β-tri-calcium phosphate (β-TCP) containing spheres [18] suggested an efficient resealing of the dentinal tubules openings as well as a repair of cracks in the enamel.

The resealing potency of polyP can be deduced from its morphogenetic properties to cells residing in the dentin, e.g., through activation of ALP and of BMP-2, two factors critically important for dentin development and growth [19,20]. The repair of enamel became suggestive due to the developed concept that the transformation of ACC to ACP is accelerated by P_i_, originating from polyP, and the subsequent phase transformation to HA (reviewed in: [12]). Recently, we could show that after inclusion of retinol into polyP-based microspheres these particles acquire the potency to induce collagen, especially those types that form fibrillar collagens, e.g., type I, III and V [18]. Since collagen, especially of type I, is known to be a major component during healing of surgical periodontal defects [21,22], a beneficial effect on the periodontal apparatus might be anticipated. Another potentially beneficial property of polyP on tooth morphology resides in the published potential of this polymer to act as an antimicrobial agent and prevent spoilage of food (reviewed in: [23,24]). To be more specific, polyP was found to interfere with growth of Gram-positive bacteria, while Gram-negative bacteria are generally more resistant [25,26]. The effect of polyP, very likely caused by selective chelation, is considerably specific and impairs the growth of *Bacillus subtilis* and *Staphylococcus aureus* and, to some extent, of *Lactobacillus plantarum* [25].

It is the aim of the present study to determine the effect of a-polyP microparticles, enriched with retinyl acetate, on repair of cracks in enamel as well as on the resealing of dentinal tubules in dentin and finally to define the effect of polyP on growth of *Streptococcus mutans*, a facultatively anaerobic, gram-positive coccus with pronounced cariogenic properties (reviewed in: [27]). The a-polyP microparticles were incorporated into a basis dentifrice/toothpaste [abbreviated “BP”], composed of the following ingredients: carboxymethylcellulose, Na-methyl-*p*-hydroxybenzoate, Na-saccharin, glycerol, Na-lauryl sulfate and water [28]; Na-fluoride and silica have not been added. Those components are frequently supplemented to commercially available toothpastes/dentifrices [29]. The results summarized in the present study show that the a-polyP-containing toothpaste efficiently repairs cracks in the enamel, reseals dentinal tubules of the dentin and finally efficiently inhibits growth of *S. mutans*; therefore we termed this formula “dentoReseal™” (abbreviated “dRs”). In the composition used here, no additional antimicrobial agent frequently used in toothpastes, e.g., triclosan (5-chloro-2-[2,4-diclorophenoxy]phenol), was added. Triclosan has been accounted to induce reactive oxygen species activation [30], but has been rated to be safe for toothpastes [31]. We report that triclosan, being a component of the commercially sold Colgate-Original toothpaste (http://www.colgateprofessional.com/products/colgate-total-advanced-toothpaste/faqs), strongly inhibits the growth of widespread bacteria, e.g., *Staphylococcus aureus* [32]. Those bacteria can develop a certain degree of resistance against triclosan. However, triclosan-containing toothpaste does not display any growth inhibitory activity against *S. mutans*; in contrast, polyP in the salt form of Zn-polyP distinctly impairs multiplication of this bacterium. The amorphous polyP-based microparticles used for the preparation of the described dentifrice were additionally supplemented with retinyl acetate to spike the mineralization-mediating and inducing activity of polyP with the gene inducing function of the retinoid. This retinoid containing polyP microparticles cause an induction of *collagen type I* gene expression, which together with the *ALP* gene is a marker for differentiation of the mesenchymal stem cells (MSC) into odontoblasts [33].

These beneficial properties of “dentoReseal™” should qualify the dentifrice as a solution for treating enamel and dentin hypersensitivity and caries, and as a morphogenetic agent supporting the reconstitution of the periodontium, especially by inducing collagen gene expression in the tissue.

## 2. Materials and Methods

### 2.1. Materials

Sodium polyphosphate (Na-polyP of an average chain of 40 phosphate units) was obtained from Chemische Fabrik Budenheim (Budenheim, Germany); retinyl acetate (syn. retinol acetate, vitamin A acetate) dissolved to ≈50% (*w*/*w*) in peanut oil (≈ 1500 U/mg) and containing dl-α-tocopherol as stabilizer was purchased from Sigma-Aldrich (#95140; Steinheim, Germany).

### 2.2. Fabrication of Ca-polyP/retinyl Acetate Microparticles

The general procedure for the preparation of the Ca-polyP/retinyl acetate microparticles was as described before [16,18], with a few modifications. At first, amorphous polyP microparticles were prepared as reported [18]. Then, 2 mL retinyl acetate oil were dissolved in 100 mL ethanol and added to 20 g of the Ca^2+^-polyP microparticles. After stirring for 3 h at room temperature (under avoidance of light), the suspension was centrifuged and the sediment was dried at 60 °C overnight. Then the particles were grinded in a Waring blender and sieved through 100 µm mesh (Retsch, Haan, Germany). The sample of microparticles, fabricated from polyP and containing retinyl acetate, was termed “a-polyP/RA-MP”.

In a separate series, 1 g of Na-polyP was added to 2.63 g of zinc chloride (#793523 Sigma-Aldrich, Steinheim, Germany) and processed as described for “a-polyP/RA-MP”. The atomic ratio of Zinc to phosphate was determined to be is 2:1, as determined by inductively coupled plasma mass spectrometry. The particles formed were termed “Zn-a-polyP-MP”.

X-ray diffraction analysis and Fourier transform infrared spectroscopic analysis were applied to verify the amorphous state of polyP [11]. The sizes of the particles range from 100 to 200 nm. The retinyl acetate content was determined using the colorimetric assay [18,34] and found to be 5.4 ± 0.3 mg per 1 g of polyP.

### 2.3. Toothpaste Test Samples

The microparticles “a-polyP/RA-MP” were blended into the basis toothpaste, composed of carboxymethylcellulose, Na-methyl-*p*-hydroxybenzoate, Na-saccharin, glycerol, Na-lauryl sulfate, 20% (*w*/*w*) Ca^2+^ carbonate (E170, CaCO_3_; Diaclean, Castrop-Rauxel, Germany) and water [28], at a concentration of 1% (*w*/*w*) (routinely) and 10% (*w*/*w*), respectively. The dentifrice paste was mixed until homogeneity and stored at ambient room temperature in an airtight, opaque container until usage. This formula was termed “dentoReseal™”, and abbreviated “dRs”-1% or “dRs”-10%. Where indicated a commercially sold dentifrice (termed “CO”) was tested in parallel. The control paste was termed “BP”. These samples were suspended in PBS (phosphate-buffered saline). The concentration indicated with the respective experiment refer to the undiluted paste sample.

### 2.4. Human Mesenchymal Stem Cells

Human mesenchymal stem cells (MSC) were isolated from normal (non-diabetic) adult human bone marrow of normal volunteers and purchased from Lonza Cologne (Cologne, Germany). Incubation was performed as described [35]. The cells were maintained in 75 cm^2^ flasks and cultivated in α-MEM (Cat. no. F0915; Biochrom, Berlin, Germany), supplemented with 20% FCS (fetal calf serum; Biochrom, Berlin, Germany) and 0.5 mg mL^−1^ of gentamycin, 100 units mL^−1^ penicillin, 100 mg mL^−1^ of streptomycin and 1 mM pyruvate (#P2256 Sigma-Aldrich, Taufkirchen; Germany). Incubation was performed in a humidified incubator at 37 °C.

For cell viability and gene expression studies the MSC cultures were inoculated with 1 × 10^4^ cells per well (48 well plates (#CLS3548; Sigma-Corning, Taufkirchen, Germany)) in a total volume of 0.5 mL. The cultures were first incubated for a period of three days in the absence of the mineralization-activating cocktail (MAC). Subsequently, the cultures were used for the cell viability studies. For the gene expression studies the cells were incubated for a total period 21 days in the presence of MAC, comprising 50 mM ascorbic acid and 10 nM dexamethasone to induce biomineralization [36]. The third component usually used in the MAC, β-glycerophosphate, was omitted since polyP has been shown to be sufficient as a phosphate supply [37].

### 2.5. Cell Proliferation/Cell Viability Assays

Quantifying cell growth/metabolic activity was performed by a colorimetric method based on the tetrazolium salt XTT (2,3-bis-(2-methoxy-4-mitro-5-sulfophenyl)-2*H*-tetrazolium-5-carboxanilide Cell Proliferation Kit II; Roche, Mannheim, Germany), as described [38]. The absorbance was determined at 450 nm and subtracted by the background values (500 nm). Routinely, the viable cells were determined after 72 h.

### 2.6. Gene Expression Studies

We applied the technique of quantitative real-time reverse transcription polymerase chain reaction (qRT-PCR) to quantitate the effect of the pastes to the MSC after the 21 day incubation in the presence of MAC. Details have been given before [39]. The following two genes for the human MSC were selected, and primers were designed against them. First, the *ALP* (*alkaline phosphatase*; NM_000478.4) Fwd: 5′-TGCAGTACGAGCTGAACAGGAACA-3′ (nt_1141_ to nt_1164_) and Rev: 5′-TCCACCAAATGTGAAGACGTGGGA-3′ (nt_1418_ to nt_1395_; 278 bp) and second the *COL I* (*collagen type I*; NM_000088) Fwd: 5′-TATGG-GACCCCAAGGACCAAAAGG-3′ (nt_1122_ to nt_1145_) and Rev: 5′-TTTTCCATCTGACCCAGGGGAACC-3′ (nt_1257_ to nt_1234_; 136 bp). The expression levels of the respective transcripts were correlated to the reference housekeeping gene *YWHAZ* (*tyrosine 3-monooxygenase*/*tryptophan 5-monooxygenase activation protein*; NM_003406) Fwd: 5’-GCTTGCATCCCACAGACTATTTCC-3’ (nt_2473_ to nt_2496_) and Rev: 5’-GGCAGACAATGACAGACCATTCAG-3’ (nt_2596_ to nt_2573_; 124 bp). The cells were extracted for RNA using the TRIzol reagent and subjected to qRT-PCR. The reactions were run at an initial denaturation of 95 °C for 3 min, followed by 40 cycles, each with 95 °C for 20 s, 58 °C for 20 s, 72 °C for 20 s, and 80 °C for 20 s. Finally, the fluorescence data were computed at the 80 °C step. The quantitative real-time PCR experiments were performed in an iCycler (Bio-Rad, Hercules, CA, USA); the mean C_t_ values and efficiencies were calculated with the iCycler software (Bio-Rad, Hercules, CA, USA); the estimated PCR efficiencies range between 93% and 103%.

### 2.7. Bacterial Studies

Two bacteria were included into the panel; first the *Staphylococcus aureus* (subsp. aureus; DSM No. 2569) and second the *Streptococcus mutans* (DSM No. 20523). Both strains were obtained from the DSMZ-German Resource Centre for Biological Material (Braunschweig, Germany). *S. aureus* was cultivated on Columbia agar supplemented with 5% horse blood (Becton-Dickinson, Le Pont-de-Claix, France [40]). The *S. mutans* was cultivated as described [41] on 5% defibrinated sheep blood agar. Cultivation was performed in an incubator (5% CO_2_).

For testing of antibacterial assay the paper disc assay was applied as described before [42,43,44]. Sterile paper discs (Whatman 3MM; Fisher Scientific, Schwerte, Germany) with a diameter of 5 mm were placed onto the Petri dishes (94 mm × 16 mm), containing the culture agar. Overnight cultures were made which gave for *S. aureus* an OD_600nm_ density of ≈3.0 and for *S. mutans* of ≈ 1.2. *S. aureus* samples were taken and diluted 1:3 with LB medium (Luria/Miller; #X968.2; Roth, Karlsruhe, Germany) giving an OD_600nm_ of ≈ 1.0; then 350 µL were plated out the plates with a Drigalski spatula (Buddeberg GmbH, Mannheim, Germany) and allow the surface to “dry” agar. For *S. mutans* 350 µL were applied undiluted onto the agar. After an incubation period of 18 h at 36 °C, the samples were inspected. A clear resolution between the dense bacterial colony regions and the bacterial growth inhibition zones around the filter discs was possible. *Escherichia coli* TOP10 (a MC1061 electrocompetent *E. coli* derivative; Invitrogen, Darmstadt, Germany) was used for a parallel series of experiments. The test samples were “BP”, “CO” and “Zn-a-polyP-MP”.

### 2.8. Tooth Samples

We used molar and premolar human teeth as sample material for treatment with experimental dentifrices. They were provided by the Institute of Functional and Clinical Anatomy, University Medical Center of the Johannes Gutenberg University, Mainz, Germany, according to the ethical guidelines of the University Medical Center Mainz. Prior to use, the specimens were cleaned from organic material by incubation in 4% sodium hypochlorite solution for 4 h. Sodium hypochlorite had been described to be a safe agent to clean surfaces of teeth from organic residual material [45]. After treatment with sodium hypochlorite the samples were thoroughly rinsed with distilled water then air dried. The cracks as well as the caries lesions seen in the teeth studied here are natural and existed already in the original teeth from the donors.

### 2.9. Cutting of the Tooth Samples

Prior to cutting, the teeth were embedded in the Technovit3040 two-component resin (Heraeus Kulzer GmbH, Wehrheim, Germany) according to the instructions of the manufacturer. Median cuts of the teeth were performed with a water cooled saw microtome, equipped with a diamond-coated inner hole saw (Leica SP1600; Leica Biosystems, Nussloch, Germany) with a layer thickness of 3 mm. After cutting the sections were cleaned with distilled water and then dried. They were stored on humidified filter paper in sterile Petri dishes at room temperature until use.

Brushing was performed with an electric toothbrush (Braun Oral-B PRO 6000; Procter & Gamble, Cincinnati, OH, USA) at 8000 rpm and 100 g force for 3 min at room temperature, as described [46]. The cut teeth were immobilized with their cut surfaces onto a glass slide and kept wet with distilled water. Then, ≈0.2 g dentifrice was spread evenly onto the respective entire enamel and dentin surfaces of both the control group and the experimental group and subsequently brushed. Routinely the samples were brushed twice a day for five days and then inspected. Where indicated, the samples were ultra-sonicated with a RK 100/H ultrasonic cleaning unit (Bandelin, Berlin, Germany) with 320 W for 1 or 5 min, as indicated. For this series of experiments, we used, because of practical considerations in a future application in the routine, only the “dRs”-1% formulation.

### 2.10. Microscopic Inspections

After sputter coating (Leica EM ACE200, Wetzlar, Germany) with gold in an argon atmosphere scanning electron microscopic (SEM) visualization was performed either with a Philips XL30 ESEM-FEG (environmental scanning electron microscope]/EDAX [energy dispersive X-ray spectroscopy] system (Philips, Eindhoven, The Netherlands) or a HITACHI SU 8000 electron microscope (Hitachi High-Technologies Europe GmbH, Krefeld, Germany). For EDX spectroscopy an EDAX Genesis EDX System attached to the scanning electron microscope (Nova 600 Nanolab; FEI, Eindhoven, The Netherlands) operating at 10 kV with a collection time of 30–45 s was used.

The light microscopic images were taken with a VHX-600 Digital Microscope from KEYENCE (Neu-Isenburg, Germany). The surface roughness of the tooth samples was measured with the software provided by the manufacturer.

### 2.11. Energy Dispersive X-ray Spectroscopy

Energy dispersive X-ray (EDX) spectroscopy was performed with an EDAX Genesis EDX System attached to a scanning electron microscope (Nova 600 Nanolab; FEI, Eindhoven, The Netherlands) operating at 10 kV with a collection time of 30–45 s. Areas of approximately 10 µm^2^ were analyzed by EDX.

### 2.12. Statistical Analysis

After finding that the respective values follow a standard normal Gaussian distribution and that the variances of the respective groups are equal, the results were statistically evaluated using the independent two-sample Student’s *t*-test [47].

## 3. Results

### 3.1. Fabrication of Ca-polyP/Retinol Microparticles

**Figure 1:**
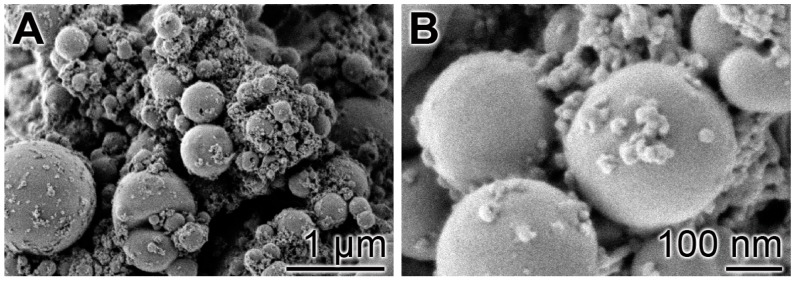
Morphology of the amorphous microparticles prepared from Na-polyP and CaCl_2_; SEM images. (**A**) Low magnification; (**B**) Larger magnification.

Following our recently described procedure [16], amorphous polyP microparticles were prepared by controlled precipitation of Na-polyP with CaCl_2_ in a weight ratio of Ca^2+^ to phosphate of 1:2. PEG [polyethylene glycol] was added during the procedure to suppress phase separation [48]. Retinyl acetate was added to the particles as described under “Materials and Methods”. In the final sample, the concentration of retinyl acetate was 5.4 ± 0.3 mg per 1 g of polyP. The morphology of the particles was close to spherical with an average size of 550 ± 120 nm (Figure 1). A quantitative EDX analysis revealed a Ca to P atomic ratio of 1 to 2 (data not shown). X-ray diffraction and Fourier transform infrared spectroscopy were used to prove that the deposits are amorphous and composed of polyP polymer chains as outlined previously [16].

### 3.2. Effect of the Microparticles on Growth of MSC

**Figure 2:**
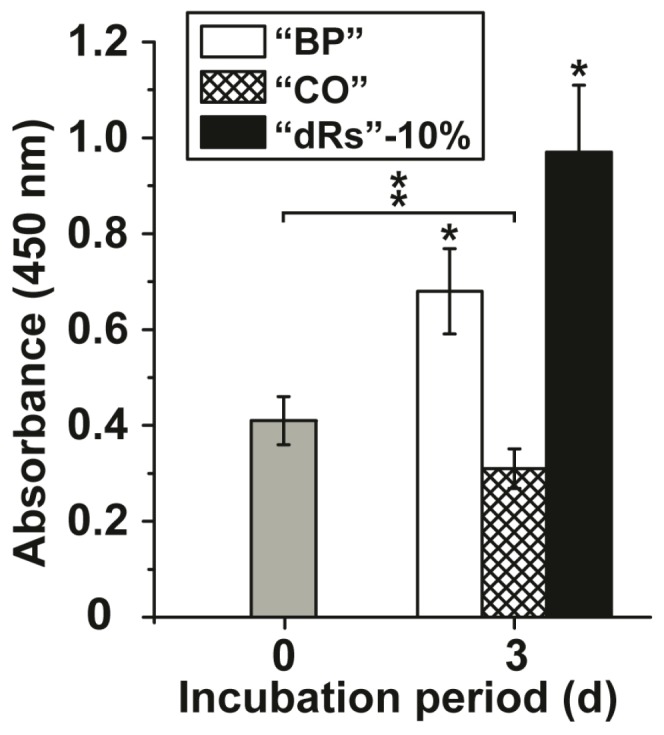
Effect of the different paste samples on viability/growth of MSC. Under otherwise identical conditions the cells were exposed to the control paste “BP” (open bars), the “CO” sample (cross-hatched) or to paste supplemented with 10% “a-polyP/RA-MP” microparticles, “dRs”-10% (filled bars). The absorbance level at time zero is given as a grey bar. A concentration of 10 µg/mL was chosen. After the three days of incubation period, the number of viable cells was determined by the XTT assay (A_450_ values). The standard errors of the means are shown (*n* = 10 experiments for each time point); *P < 0.05. The significant increase was determined pairwise between the absorbance at time 0 and “BP” as well as “dRs”-10% after the three-day incubation; * *p* < 0.05. The significant decrease between the controls at time 0 (filled in grey) and “CO” after three days is marked as ** *p* < 0.05.

The effects of the microparticles on growth of MSC were tested in the XTT metabolic analysis assay. The concentration added to the cultures was 10 µg/mL (concentration with respect to the applied paste). After a short incubation period of 3 h the absorbance was determined and found to be 0.41 ± 0.051 absorbance units at 450 nm (Figure 2); set to time 0. After a three-day incubation the absorbance in the assays with the test samples was determined again. In the control assay, the base paste “BP”, the absorbance increased significantly to 0.68 ± 0.089 absorbance units and in the paste supplemented with 10% microparticles containing retinyl acetate (“a-polyP/RA-MP”) and termed “dRs”-10% to 0.97 ± 0.14. If the cells were incubated in the presence of the lower concentration polyP microparticles paste (“dRs”-1%) the increase in the absorbance units was smaller, but still significant, with respect to the controls (0.53 ± 0.061 absorbance units; data not shown in Figure 2). In contrast, the “CO” sample was significantly inhibitory, as can be deduced from the decrease of the absorbance to 0.31 ± 0.048 (Figure 2).

**Figure 3:**
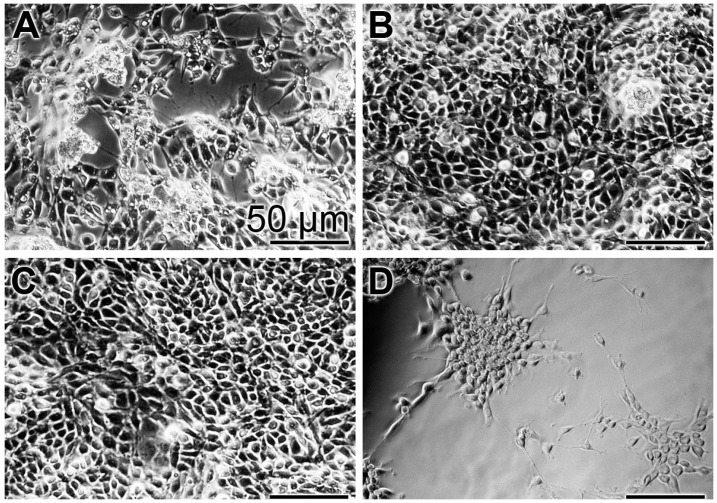
Density of MSC in assays exposed for: (**A**) the short incubation period of 3 h (set to time 0) to the base paste “BP”; or for three days to “BP” (**B**); to the paste with 10% “a-polyP/RA-MP” microparticles, “dRs”-10% (**C**); and to the “CO” formula (**D**). Light microscopy.

In order to substantiate the results from the XTT colorimetric assay, images were taken with an optical microscope (Figure 3). At time 0, a low density of cells can be imaged (Figure 3A). After the three-day incubation with the “BP” control base paste or the paste containing the polyP-containing microparticles, “a-polyP/RA-MP”, the “dRs”-10%, the cell layer is close to dense (Figure 3B,C), while only scarcely cells are seen on the bottom of the assays exposed to “CO” (Figure 3D).

### 3.3. Alteration of Gene Expression in Response to the Different Paste Formulae

**Figure 4:**
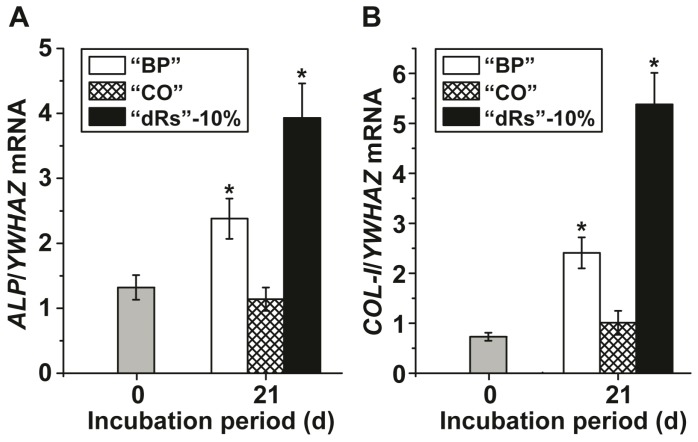
Steady-state expression of the: (**A**) *ALP*; and (**B**) *COL-I* genes in MSC, cultured in the presence of the mineralization activation cocktail, MAC, after an incubation period for 21 days. Immediately after seeding the cells the expression ratio for the *ALP* gene or of the *COL-I* gene versus the *YWHAZ* house-keeping gene was determined (time zero); grey bars. The MSC were incubated with 10 µg/mL of “BP” (open bars), “CO” (cross-hatched) or “dRs”-10% (filled bars). After incubation the cells were harvested and the RNA was extracted and subsequently subjected to RT-qPCR analysis. The expression values are given as ratios to the reference house-keeping gene *YWHAZ*. The results are means from five parallel experiments (* *p* < 0.01).

Gene expression studies were performed to assess the effect of the three different paste formulations on MSC by application of the qRT-PCR technique. After incubation of the MSC for 21 d in the presence of MAC the cells were harvested and the steady-state-expression transcript levels of the *ALP* and of *collagen type I* (*COL-I*) genes were determined. The data, summarized in Figure 4 show that during the incubation period the transcript level for *ALP* increases significantly in cultures exposed to the paste “BP” from 1.32 ± 0.19 expression units, with respect to the expression of the housekeeping gene *YWHAZ*, to 2.38 ± 0.31. Even higher is the expression level in cells, incubated with the “dRs”-10% paste; there the level reaches 3.93 ± 0.53 (Figure 4A); the increase of the “dRs”-1% is likewise higher with 3.48 ± 0.49 (data not shown in the figure). Under otherwise identical condition, the amount of transcript in “CO”-treated cells changes only non-significantly.

A similar expression pattern is seen if the *COL-I* level is monitored (Figure 4B). While again, the expression level of this gene increases significantly after exposure to “BP” (from 0.73 ± 0.08 to 2.41 ± 0.31 expression units) or to “dRs”-10% (to 5.38 ± 0.63), and to “dRs”-1% (to 3.70 ± 0.51), respectively, no change is seen in cells treated with “CO”.

### 3.4. Antibacterial Activity

**Figure 5:**
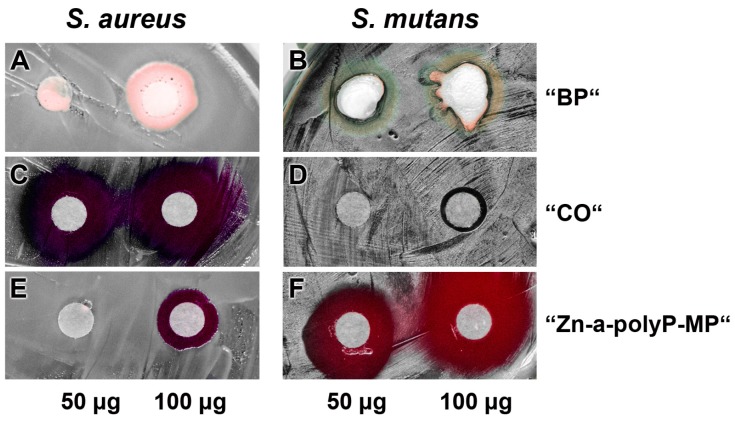
Antibacterial activity testing. Applying the conventional filter paper disc assay, the effect of: the base paste “BP” (**A**,**B**); the commercially available paste “CO” (**C**,**D**); and the “Zn-a-polyP-MP” (**E**,**F**). The quantities of the respective compounds/materials added to the discs are indicated. The culture dishes were incubated with *S. aureus* and *S. mutans*.

The pastes were studied for the (potential) antibacterial activity using the agar diffusion method (disc variant), as described under “Materials and methods” (Figure 5). The experiments reveled that the base paste “BP” (Figure 5A,B) displays no activity against both the *S. aureus* and *S. mutans*, while the “CO” pastes was very inhibitory against *S. aureus* but hardly against *S. mutans* (Figure 5C and D). In contrast, the microparticles, formed from Na-polyP and ZnCl_2_, “Zn-a-polyP-MP” (Figure 5E and F), cause a very strong inhibition of *S. mutans*, but are only marginally active against *S. aureus*. In addition, it was determined that the “CO” paste was very strongly active against *Escherichia coli*, while “dRs”-1% or “dRs”-10% was not active against this Gram-negative bacterium (data not shown).

### 3.5. Occlusion of Tooth Damages

**Figure 6:**
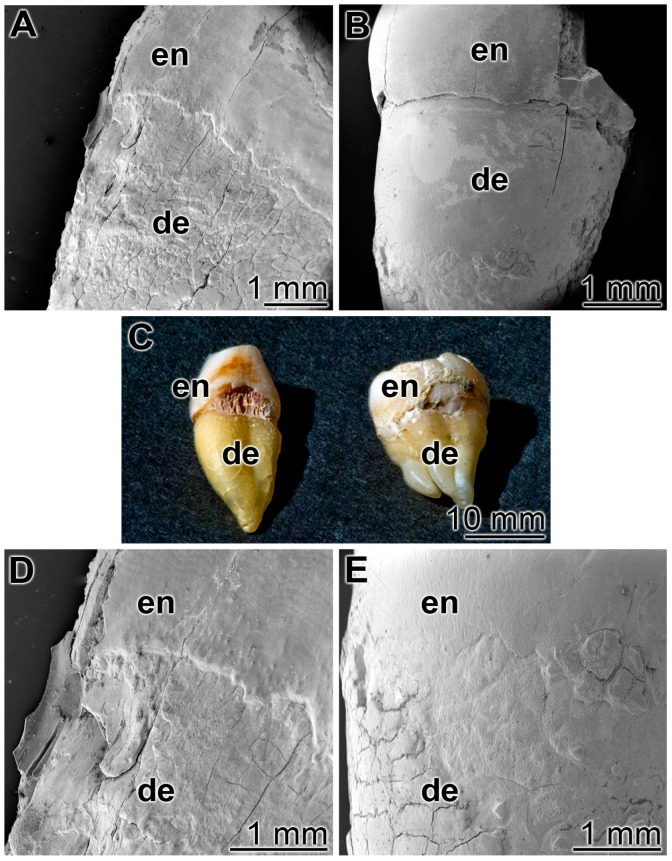
Treatment of the cut teeth either with: (**A**,**D**) the control paste “BP”; or (**B**,**E**) the “dRs”-1% polyP-containing paste. (**C**) Human teeth were cut and immobilized with the cut surfaces onto a glass slide. The enamel (en) and dentin (de) regions are marked. SEM (**A,B,D,E**) or light microscope (**C**) inspections.

As outlined under “Materials and methods” human teeth were cut in median directions and immobilized with the cut surfaces onto a glass slides (Figure 6C). Then the specimens were brushed twice a day for five days, for 3 min each, with ≈0.2 g of the respective dentifrice. Both the enamel and the dentin regions were inspected (Figure 6). Already at a low magnification it is apparent that after a five-day treatment with the control “BP” paste the damages in the enamel and the dentin regions, the breaks, cracks or fissures remained (Figure 6A,D), while in the “dRs”-1% paste a smooth surface layer became visible (Figure 6B,E). The experiments had been performed, in parallel, with five tooth samples each; representative images are given. For the occlusion and re-sealing studies, we used the lower concentration polyP in the “dRs”-1% paste because of practical considerations in the future (potential) routine application.

### 3.6. Repair of Carious Tooth Lesions in the Enamel

**Figure 7:**
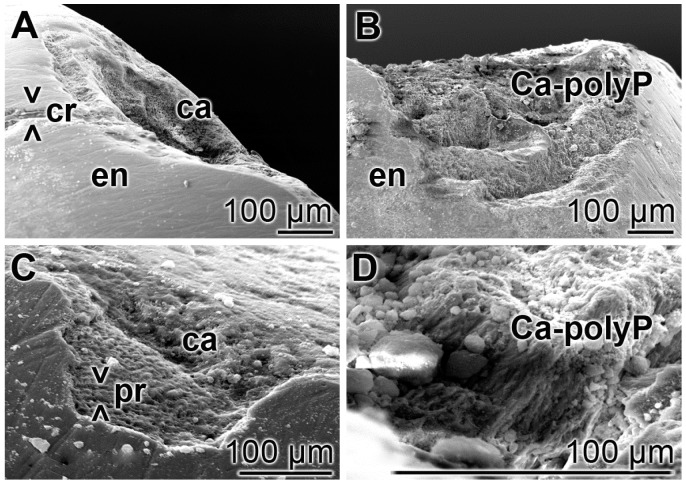
Treatment of the carious tooth lesion damages in the enamel region; SEM. The tooth sections were treated either: (**A**,**C**) with the control paste “BP”; or (**B**,**D**) with “dRs”-1% twice a day for five days. In the control “BP” samples, cracks (> < cr) remained and the deep carious lesions uncover the separately existing enamel (en) prisms (> < pr). In contrast, in the “dRs”-1%-treated specimens no cracks could be detected in the smooth surface and the dental carious cavities (ca) are filled with amorphous Ca^2+^-polyP (Ca-polyP), originating from the “dRs”-1% paste.

Teeth sections, containing carious lesions in the enamel were brushed twice daily for five days with “BP” or with “dRs”-1% and then inspected by SEM; five parallel tooth samples had been included into the study. In the control samples i.e., treated with “BP”, the existing cracks and especially the carious lesions remained (Figure 7A,C) as in the untreated controls. In the relatively smooth surface, the cracks are visible, while in the extensive carious lesions, the rows of the separated enamel prisms became overt. However, in the “dRs”-1% treated samples (*n* = 5), all crack damages are filled with paste material; the carious lesions became filled with the “a-polyP/RA-MP” component of the “dRs”-1% paste (Figure 7B,D). The elastic modulus of the repaired lesion was determined to be approximately 320 ± 181 MPa, about 20% to those values determined for enamel [49] (to be published).

**Figure 8:**
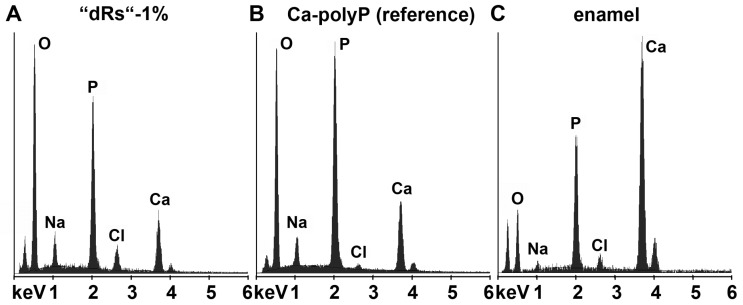
Energy-dispersive X-ray analysis plot obtained for the deposits in a caries cavity, that was brushed for (**A**) five days with “dRs”-1%. In comparison, the spectra for: (**B**) the “a-polyP/RA-MP” (Ca-polyP [reference]) material added to the “dRs”-1% paste; and (**C**) enamel are also given.

In order to clarify that within the carious tooth lesions polyP has been deposited a quantitative EDX analysis was performed. From published data it is known that the enamel has an calcium: phosphorous (Ca:P) atomic ratio of 1.5 to 1.9 [50] and Ca-polyP (microparticles) a ratio of 0.62 [16]. EDX analysis performed here revealed that the Ca:P ratio for the “a-polyP/RA-MP” used in the “dRs”-1% paste amounts to 1.53, while the deposits within the carious tooth lesions show a value of 1.62; in contrast, the respective Ca:P ratio for enamel was determined to be 1.7 (Figure 8). This finding indicates that the deposits precipitated within the caries lesion are Ca^2+^-polyP.

### 3.7. Repair of Cracks in the Enamel Region

**Figure 9:**
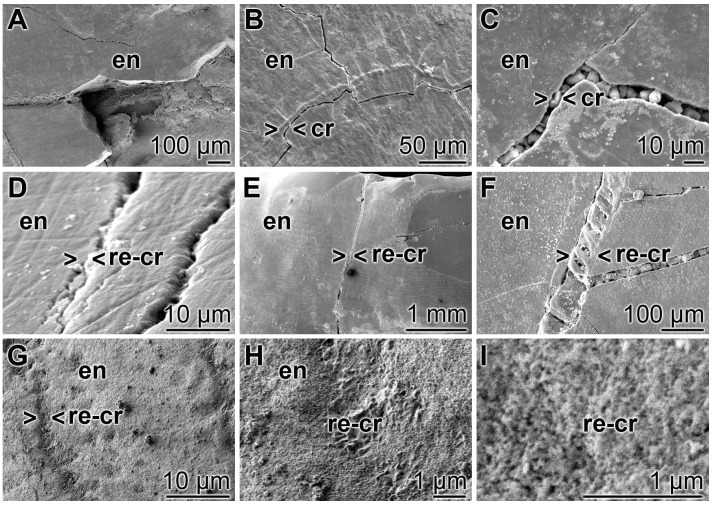
Closure of the cracks within the enamel region by polyP; SEM: (**A**–**C**) Treatment of the tooth samples with “BP”. The crack damages (cr) are constantly seen; (**D**–**F**) Brushing of the teeth with “dRs”-1% results in a repairing of the cracks (> < re-cr) within the enamel region (en); (**G**–**I**) At a higher magnification, the globular, spherical microparticles, originating from the polyP microparticles in the “a-polyP/RA-MP” sample that were fabricated into the “dRs”-1% paste become visible.

**Figure 10:**
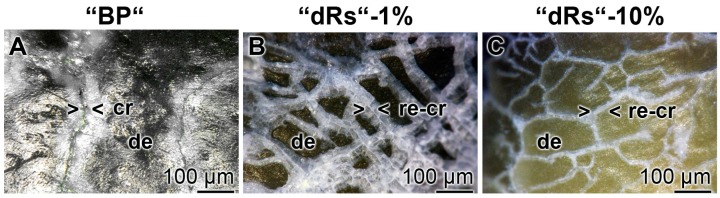
Sealing of the cracks within the dentin region; light microscopic images. The samples were treated with: (**A**) “BP”; (**B**) “dRs”-1%; or (**C**) “dRs”-10% for five days. In contrast to the samples treated with “BP”, where the cracks (cr) remained within the dentin (de) region (**A**), those damages became repaired (re-cr) after treatment: with “dRs”-1% (**B**); or with “dRs”-10% (**C**). The scale bars in all images measure 100 µm.

A closer inspection of the cracks within the enamel discloses that after treatment of the teeth with “BP” none of the damages are sealed. The cracks are visible throughout the enamel (Figure 9A–C). In contrast, if the enamel was brushed with “dRs”-1% for the same period of time the cracks are partially (or almost completely) sealed (Figure 9D–F). At a higher magnification it becomes apparent that the deposits introduced by the “BP” paste into the cracks appear as µm-sized particles that are completely separated from the surrounding tooth material (e.g., Figure 9C). Inspecting the SEM images taken from “dRs”-1%-brushed tooth specimens, small gaps at the rims of the sealing material (“dRs”-1% paste) are visible (Figure 9D–F). Those clefts are not seen in images from specimens, likewise treated with “dRs”-1% paste, but inspected with the light digital microscope (see Figure 10). We consider these fissures as artefacts that are formed during SEM inspecting by the radiation of high accelerating voltages of 15–20 kV. The material deposited into the cracks after brushing with “dRs”-1% appears—at high magnification—as globular, spherical microparticles “a-polyP/RA-MP” (Figure 9G–I).

### 3.8. Resealing of Cracks and the Surface Roughness of the Closed Cracks

**Figure 11:**
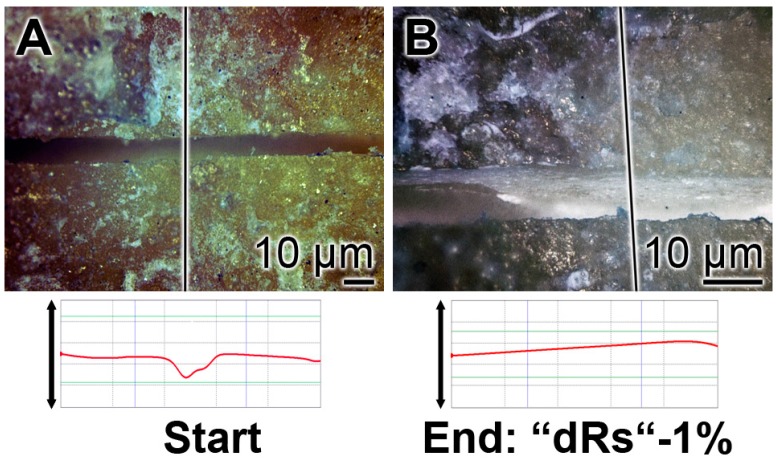
Treatment of one crack with “dRs”-1%: (**A**) start; and (**B**) termination of the five-day treatment with the “dRs”-1% paste.

The property of the “a-polyP/RA-MP”, included into the “dRs”-1% paste, to seal the cracks in the enamel is visible in the dentin region (Figure 10). Using the “BP” base paste the cracks remained after the five-day treatment period (Figure 10A). In contrast, if those samples were treated with “dRs”-1% (Figure 10B) or “dRs”-10% paste (Figure 10C) all cracks are sealed within the dentin region. In comparison, the surface textures of the same tooth at the start and after the termination of the treatment with the “dRs”-1% paste are shown in Figure 11A,B.

**Figure 12:**
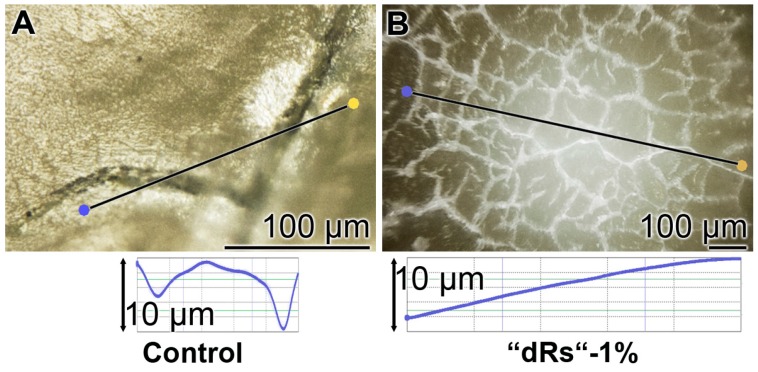
Surface morphology of the tooth dentin specimens after a five-day treatment with: (**A**) the “BP” paste; or (**B**) the “dRs”-1% dentifrice. The images reflecting the surface roughness of “BP” paste-treated crack areas (**A**) versus the one of an area treated with “dRs”-1% dentifrice (**B**) which are shown in the lower panels.

The surface roughness of those closed cracks are assessed using the Keyence light microscope and applying the software designed for it. The determinations revealed that the cracks within the dentin region, using the respective tooth specimen, are deep and measure ≈ 10 µm (Figure 12A). After treatment of the dentin sample with “dRs”-1% paste, a smooth surface is recorded (Figure 12B). This finding indicates that the surface of the “dRs”-1%-treated specimen is not rough at all but comparably smooth (Figure 12B [lower panel] versus Figure 12A [lower panel]).

### 3.9. Stability of the Sealing by “dRs”-1%

**Figure 13:**
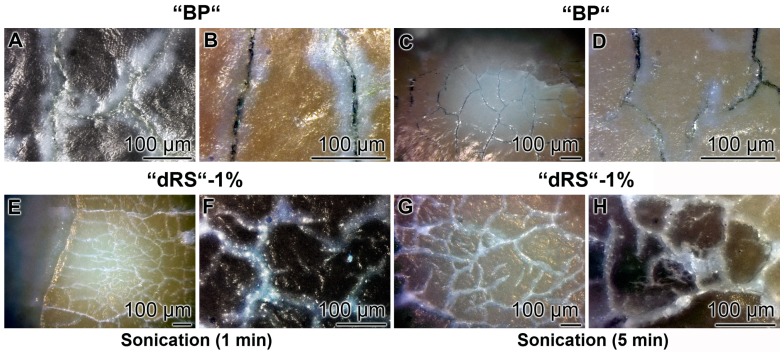
Resistance of the “dRs”-1%-mediated sealant against sonication. The teeth samples were brushed either: with “BP” (**A**–**D**); or with “dRs”-1% (**E**–**H**). Subsequently, the samples were either sonicated: for 1 min (**A,B,E,F**); or for 5 min (**C,D,G,H**). The lengths of the scale bars refer to 100 µm.

The stability of the occlusion of the cracks within the dentin was determined by sonication of the samples. The teeth were brushed for five days with the “BP”, in the absence of polyP, or the paste supplemented with “a-polyP/RA-MP”, the “dRs”-1% (Figure 13). The distinct cracks in the dentin regions remained in those samples which were treated with “BP” after a sonication for both 1 and 5 min (Figure 13A–D). Very much in contrast are the images after sonication of the teeth treated with “dRs”-1%. After a sonication period of 1 min and even 5 min the dental sealant remained unchanged (Figure 13E–H).

### 3.10. Occlusion of Exposed Dentinal Tubules

**Figure 14:**
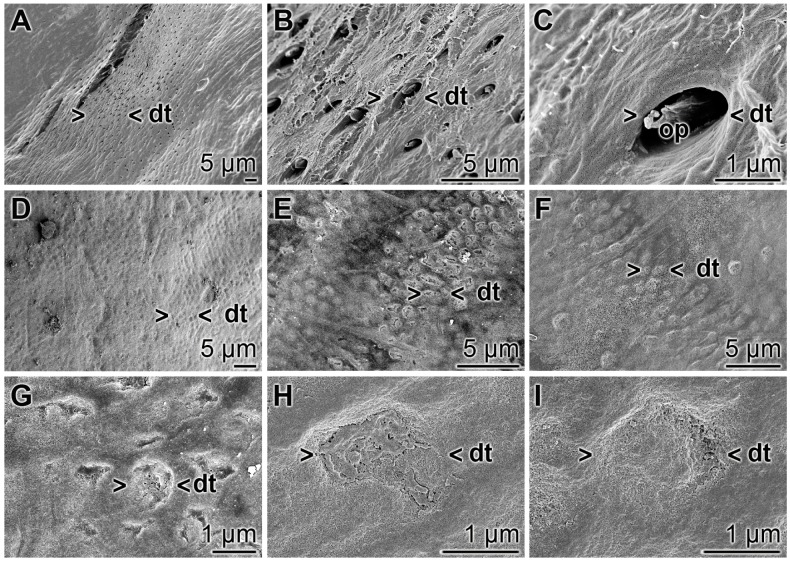
Occlusion of the dentinal tubules by “dRs”-1%; SEM: (**A**–**C**) Untreated dentin zone with openings of dentinal tubules (dt). Frequently the odontoblastic processes (op) are seen. (**D**–**I**) Sealing of the dentinal tubules (dt) after a five-day brushing treatment, twice daily, with “dRs”-1%.

The occlusion effect of the polyP microparticles containing dentifrice, “dRs”-1%, towards dentinal tubules was studied by SEM analysis (Figure 14). The dentinal tubules have a size of ≈ 1.5 to 4 µm (Figure 14A,B) and show inside of them the odontoblastic processes (Figure 14C). If the teeth specimens were treated with “dRs”-1% for five days those openings of the dentinal tubules were sealed/re-sealed (Figure 14D–I). It is amazing that even after the short treatment time of five days most of the openings are covered, e.g., as in Figure 14G–I.

## 4. Discussion

In an abiotic, non-aqueous environment, along with non-harsh physical and chemical conditions, the crystalline bone mineral HA, e.g., in bone, dentin and enamel, is a stable structure [51]. However, under physiological, biotic conditions, HA is amazingly unstable and prone to continuing catabolic and subsequent anabolic transformations [52]. This metabolism is dependent on changes in the physical, chemical and biological/hormonal conditions. In addition, some ions, e.g., the anion fluoride, rapidly exchange for hydroxyl ions on the surface of HA (Ca_10_(PO_4_)_6_OH_2_) under formation of fluoroapatite (Ca_10_(PO_4_)_6_F_2_) [53]. Focusing on the direct bone surface the remodeling period, reflecting osteoclastic resorption and osteoblastic formation, amounts to about 200 days in humans [54]. This high turnover of the bone mineral is readily conceivable in view of the fact, that 99% of the body’s calcium, 85% of phosphorus, and 65% percent of total body magnesium are trapped within the bones (see: [55]). Bone resorption, mediated by osteoclasts, is initiated by the secretion of hydrogen ions and lysosomal enzymes, in particular of cathepsin K. Already the slight shift of the pH in the HA environment from the physiological value of pH 7.45 to 6.7 causes a increase of the osteoclast-mediated resorption of the bone surface from 1000 to 8000 µm^2^ [56]. While the crystalline HA in the abiotic, non-aqueous environment at neutral pH is inert it is readily dissolved in the resorption lacuna with a pH of ≈4.5–4.8 [57]; during this dissolution process of the inorganic bone matrix not only calcium and phosphate ions, but also bicarbonate ions are released. The latter finding, the production of carbonate/bicarbonate strongly supports our bio-seed view on bone-HA formation, a process driven by the carbonic anhydrase(s) [10,58].

The physiological polymer polyP, formed abundantly in animal/mammalian cells (see “Introduction”), causes a shift of pH down to pH 4–5 after dissolution in water due to a hydrolytic cleavage of the phosphoanhydride bonds. It is especially the amorphous polyP that more rapidly hydrolyzes and by that efficiently lowers the pH environment [59]. The existing evidence suggests and the chemical reaction conditions strongly indicate that polyP in the body exists as a Ca^2+^ salt in the amorphous state [15]. Recently, we succeeded in fabricating Ca^2+^-polyP salt complexes, nanoparticles and microparticles that are considerably stable and elicit the well-established morphogenetic properties, e.g., the induction of the steady-state expression of the genes encoding *BMP-2* and *ALP* (reviewed in: [10,13]). Those polyP particles were found to allow the preparation of microspheres that can be potently used as implant material for bone defects [18]. If those polyP particles are supplemented with retinol they efficiently induce a series of *collagen* types as well [18]. In previous in vitro [37] as well as in vivo studies [60] it has been unequivocally shown that polyP in the non-particle bound form acts anabolically on bone mineral formation. However, the nanoparticles/microparticles fabricated from Ca-polyP display a stable form for this polymer to be embedded not only in implant material to be used for regeneration of bone defects but also into dentifrice as more stable carriers.

**Figure 15:**
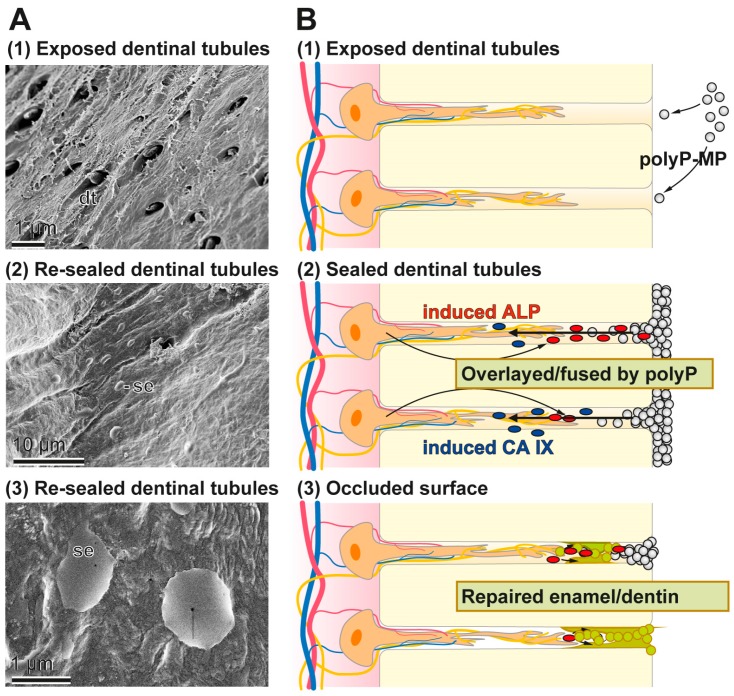
Occlusion of the dentinal tubules by the polyP-based and microparticle-formulated “dRs”-1% paste. In the first step the microparticles adsorb to the HA surface where in the second step an ALP-driven hydrolysis of the polyP within the microparticles takes place, resulting in a lowering of the pH. After this resealing phase the dentinal tubules are occluded by a genuine repair process. The different phases are illustrated: (**A**) by the respective SEM images; and (**B**) by the corresponding sketches.

In the present study we prepared Ca-polyP microparticles that are enriched with the more stable retinyl acetate instead of retinol, since it also contains dl-α-tocopherol as stabilizer [61]. The polyP-based toothpaste fabricated here, the “dRs”-1% “dentoReseal™” efficiently resealed the cracks and the carious damages of the human teeth. Retinyl acetate was included into the toothpaste in order to enrich it with a collagen-inducing morphogen [18]. Such an activity is advisable to be present in those toothpastes which should elicit an ameliorating effect on periodontitis lesions. It is amazing that even a five-day application/brushing of the specimens, twice daily, causes (almost) complete filling and occlusion of the cracks. Such a filling of the splits can likely be achieved by other, hitherto introduced dentifrices including also the HA-containing pastes. However, unique appears the property of the “dRs”-1% paste even to be resistant against a short sonication by a high power (320 W) sonicator. Those HA-containing dentifrices contain crystalline HA particles that surely cannot form such an intimate bonding at physiological pH-neutral conditions. The deposits formed onto the carious tooth holes that have been formed after the five-day treatment with “dRs”-1% is still amorphous Ca-polyP in nature and not crystalline HA. The EDX spectra clearly support this finding. As a consequence a longer application period of “dRs”-1% is advisable, allowing the transition of Ca-polyP to the HA phase under physiological oral mucosal pH value (6.8 to 7.4) conditions in healthy saliva [62]. The growth of HA from amorphous Ca-polyP will proceed under near neutral pH values by surface controlled mechanism(s) and advances from a crystalline seed phase, such as octacalcium phosphate and apatite due to the growth of microcrystalline deposits [63]. The implications of this finding, the polyP-based and microparticle-formulated “dRs”-1% paste, to bind and reseal firmly damages of the enamel and dentin, are that “dRs”-1% can be beneficial in a straightforward solution for dental hypersensitivity and other dentin and enamel caries-like lesions produced by mechanical impact as well as microbial destructions. A schematic outline of the resealing of the exposed dentinal tubules by the polyP-microparticles in “dRs”-1% is given in Figure 15. The Ca-polyP ingredient is layered on top of the HA surface and might protrude to some extent into the tubules. After the overlay phase a fusion stage might follow during which the Ca-polyP polymers undergo degradation by the ALP under lowering the pH of the environment. During the lowering of the pH, and driven by the activity of the carbonic anhydrase IX, an acceleration of the physiological mineralization of the Ca-phosphate deposits will proceed during which first the amorphous Ca-carbonate bio-seeds and subsequently the Ca-phosphate minerals are synthesized.

The dentifrice, developed here “dRs”-1%, also offers two more additional and beneficial properties. First, in the present study, we did not include into the Ca-polyP microparticles any additional antibacterial component, e.g., triclosan (see “Introduction”). It is shown for the first time that the microparticles formed from polyP and ZnCl_2_, the “Zn-a-polyP-MP”, comprises antibacterial effect towards the main bacterium causing pronounced cariogenic damages, *S. mutans*, a facultatively anaerobic, gram-positive coccus. Such potency has not been described for triclosan, very often used in commercial dentifrices, e.g., Colgate-Original. It might be highlighted that a global antibacterial property, such as those described for triclosan, is broad and hence not specific [64]. It should be stressed that besides those caries-causing bacteria, physiologically existing microorganisms are present in the healthy oral cavity to which a physiological role can be attributed [65]. In addition, the anti-microbial potential triclosan has been detected in breast milk, urine and plasma, with levels which might cause endocrine disruption in mammals [66]. In the present study, a mammalian cell-toxic effect has been identified for the triclosan-containing Colgate-Original paste. Therefore, the new toothpaste formula “dentoReseal™”, prepared with “dRs”-1%, will be supplemented with “Zn-a-polyP-MP”.

Finally, the present dentifrice, composed of the polyP-based “dRs”-1% “dentoReseal™” dentifrice, which did not inhibit MSC cell growth under the conditions used, was supplemented with retinyl acetate, to strengthen the morphogenetic activity elicited by polyP, and to provide the formula with the potential to induce collagen, as recently demonstrated [18]. Indeed, it could be demonstrated by qRT-PCR that those microparticles induce in MSC the steady-state expression of *collagen type I*. This fibrillar structural protein is essential for the reconstitution of the periodontium, especially after caries damages [67].

## 5. Conclusions

**Figure 16:**
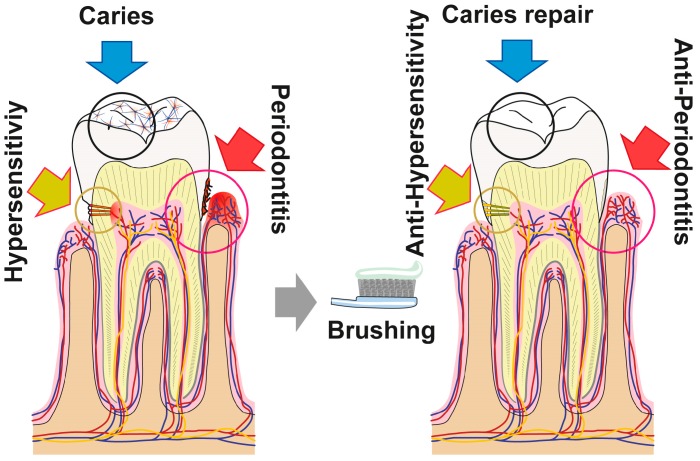
The polyP-containing formula “dRs”-1% (“dentoReseal™”) comprising trifunctional activity: First, resealing of cracks within the enamel and dentin regions of the teeth: ameliorate hypersensitivity by acting anti-sensitively. Second, filling out of carious damages: anti-cariously via remineralization processes. Third, the new formula is proposed to repair periodontitis lesions via induction of tissue repair. The first and the second modes of action are caused by the direct function of polyP, while the third property is due to the concerted morphogenetic activity elicited by this inorganic polymer (induction of the *ALP* gene) and the retinyl acetate (upregulation of the steady-state-expression of *collagen type I*).

The data summarized in the present report show that the novel dentifrice, which contains amorphous polyP and retinyl acetate as the major bioactive components, executes three activities (resealing of cracks/fissures within enamel/dentin; filling carious cavities; and remineralization) beneficial for the repair and prevention of carious damages in the dentin as well as enamel of teeth. As sketched in Figure 16 the damages, such as enamel defects and those caused by dental caries, can be efficiently and sustainably resealed by the polyP component. The deposits formed from those microparticles are firmly attached to the HA surface and presumably—at least transitionally—ameliorate dental pain etiologically originating from those damages. Moreover, “dRs”-1% might be effective in the desensitization and of managing dental hypersensitivity. Besides of this mineral deposition property of polyP, its inducing property on genes, involved in bone formation, like ALP, should be mentioned. This protein is required for the mineralization onto odontoblastic processes and for the enzymatic hydrolysis of polyP. The latter reaction might trigger the transformation of the existing crystalline HA as well as the amorphous Ca-polyP deposits to mutual and fused mineralic crystalline patches (Figure 16).
